# Measurement of Real-Time Serotonin Dynamics from Human-Derived Gut Organoids

**DOI:** 10.1021/acs.analchem.4c06033

**Published:** 2025-02-26

**Authors:** Bettina Bohl, Yuxian Lei, Gavin A. Bewick, Parastoo Hashemi

**Affiliations:** † Department of Bioengineering, Imperial College London, South Kensington, London SW72AZ, United Kingdom; ‡ Diabetes and Obesity Theme, School of Cardiovascular and Metabolic Medicine and Sciences, Faculty of Life Sciences and Medicine, King’s College London, London SE1 1UL, United Kingdom; § Diabetes Endocrinology and Obesity Clinical academic Partnership Kings Health Partners, London SE1 9RT, United Kingdom

## Abstract

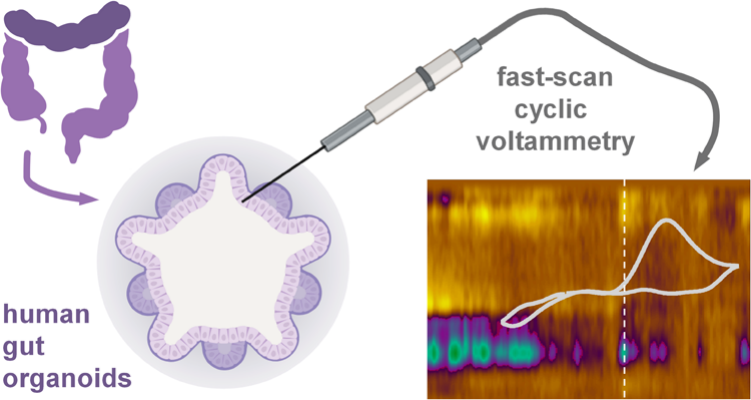

The importance of the gut in regulating the brain–body immune axis is becoming increasingly evident. Interestingly, the brain and gut share many common signaling molecules, with serotonin being one of the most notable. In fact, the gut is the primary source of serotonin in the body. However, studying serotonin dynamics in a human-specific context remains a challenge. Human stem cell-derived models provide a promising avenue for studying signal transmission in well-controlled, *in vitro* environments. In this study, we report the first fast-scan cyclic voltammetry (FSCV) measurements of serotonin signaling in a newly developed enterochromaffin cell (ECC)-enriched gut organoid model. First, we characterize the stem cell-derived gut organoids and confirm they are enriched with ECCs, the key cell type responsible for producing and releasing serotonin in the gut. We then optimize an *in vitro* buffer that maintains cell viability while supporting FSCV measurements. Using this system, we detect spontaneous release events, which increase in frequency and amplitude following stimulation with forskolin (FSK) and 3-isobutyl-1-methylxanthine (IBMX). Finally, we confirm the identity of the signal as serotonin using a selective serotonin reuptake inhibitor (SSRI), which significantly delayed the reuptake profile. Our study introduces the first real-time measurement of serotonin signaling in a human-derived gut model. We believe this system will be essential for future research on serotonin’s role in the gut and for potential novel drug target identification.

## Introduction

The gut is a critically important area of biological study due to its complex and dynamic functions. Key players in this regulation are the hormone and neurotransmitter producing enteroendocrine cells that are scattered throughout the gut epithelium. These cells act as central communicators, translating luminal, neural, and immune signals into functional responses, locally and body-wide. They regulate a broad spectrum of physiological processes, including maintaining metabolic homeostasis, digestion, and gut–brain communication. Disruption of this signaling has been linked to conditions such as irritable bowel syndrome (IBS)^1−3^ and mental health disorders.^4,5^ The gut and brain are connected through a bidirectional immune axis, making gut inflammation and depression well-known comorbidities.^6^ Interestingly, several neurotransmitters that regulate mood in the brain also exist at high concentrations in endocrine cells in the gut. For instance, enterochromaffin cells (ECCs) are a subset of the enteroendocrine population and produce serotonin.^7^

In the gastrointestinal tract, serotonin regulates peristalsis, motility, fluid and mucus secretion, and nutrient absorption.^8−10^ Elevated serotonin levels are observed in the colon and ileum during inflammatory bowel disease (IBD) and in response to bacterial stimulation,^11,12^ while disruptions in serotonin signaling have also been implicated in IBS and gastrointestinal motility disorders.^1^ Beyond local effects on the gastrointestinal (GI) tract, serotonin plays a crucial role in the gut–brain axis, where it is thought to modulate mood, stress response, and neuro-gastroenterological disorders.^13^ Dysregulation of serotonin signaling may contribute to these conditions by altering immune cell responses, the gut microbiome, and epithelial barrier integrity.^14−17^ Targeting serotonin pathways presents potential novel therapeutic opportunities for many GI disorders. However, there is a critical need to develop more physiologically relevant and specific tools for studying serotonin’s role in the gut.

Over the past decade, electrochemical techniques have offered the first insights into the dynamics of serotonin secretion in the gut. Patel et al. utilized boron-doped diamond electrodes to simultaneously measure serotonin and melatonin from the mucosa in the rabbit ileum, showing important mechanistic differences between the regulation of the two modulators.^18^ Amperometric measurements in single human neuroendocrine carcinoid BON cells revealed that 80% of vesicular contents are released during signaling.^19^ In both primary cell preparations and cancer cell lines, amperometry demonstrated that although these cells express proteins related to neuronal release machinery, their signal kinetics resemble those of endocrine rather than neuronal signaling.^20^ Yeoman et al. investigated serotonin signaling in the mouse colon and ileum and discovered regional differences were due to variations in serotonin autoreceptor and serotonin transporter density.^21^ The development of flexible electrochemical probes enabled the first *in vivo* amperometric measurements in mice, with minimal impact on tissue integrity or function.^22^ Recently, fast-scan cyclic voltammetry (FSCV) has been applied to *ex vivo* gut slices for the first time, revealing the simultaneous release of serotonin and melatonin.^23^ FSCV can differentiate between various chemical compounds—such as neurotransmitters (*e.g.*, serotonin, dopamine), hormones, or metabolites because of cyclic voltammograms (CVs) with discrete oxidation and reduction potentials afforded by the technique.

Human-derived models offer an opportunity to study human-specific phenomena in well-controlled *in vitro* environments. The most accessible approach to achieving this is through stem cell-derived models. Despite the significance of ECCs, studying human ECC biology has been challenging due to their sparse distribution and the difficulties associated with isolating and maintaining primary human ECCs in culture. Much of our current knowledge stems from murine models, where fluorescent reporters have enhanced our understanding of ECC distribution, differentiation, and function.^24,25^ However, interspecies differences underscore the need for a better understanding of human biology. The recent convergence of organoid technology and CRISPR-Cas9 gene editing has transformed the field. By culturing organoids and introducing fluorescent labels via CRISPR-Cas9, we can now visualize and study human ECCs in unprecedented detail. However, such models have not been widely probed by using electrochemistry.

In this article, we generated human CHGA-mNeon-expressing colonoids using CRISPR–Cas9-mediated homology-independent transgenesis (CRISPR-HOT). In this context, CHGA (chromogranin A) serves as a marker for ECCs.^26^ We began by confirming that colonoids contained the necessary biochemical machinery for synthesizing serotonin. Next, we optimized an *in vitro* buffer to ensure compatibility between the organoid culture conditions and FSCV measurements. We subsequently measured spontaneous events and observed CVs corresponding to serotonin, which increased in frequency and amplitude when stimulated with agents that activate cyclic adenosine monophosphate (cAMP) signaling, forskolin (FSK), and 3-isobutyl-1-methylxanthine (IBMX). Finally, we verified the identity of the signal using a selective serotonin reuptake inhibitor (SSRI), an agent that inhibits the serotonin transporters (SERTs). The SSRI significantly slowed the reuptake profile of the signal.

In summary, we present a human gut organoid model that can be used to measure real-time secretion of serotonin from ECCs using FSCV. We believe this model will be invaluable for future studies on the roles of serotonin in human gut health and disease.

## Methods

### Sample Collection and Cell Culture

Human colon organoids (colonoids) were derived from colonoscopy biopsies of a 50 year-old male patient (unique identifier FG) from Kings College Hospital NHS Foundation Trust. Briefly, the biopsies were rinsed in ice-cold phosphate-buffered saline (PBS; D8537, Sigma-Aldrich) and twice in 10 mM 1,4-dithiothreitol (DTT; 10197777001, Sigma-Aldrich) for 5 min at room temperature. To release crypts, tissue was incubated by rotating in 8 mM ethylenediaminetetraacetic acid (EDTA) (15575–038, Invitrogen) in PBS for 1 h at 4 °C and shaken vigorously in cold PBS. The supernatant was centrifuged for 3 min at 400 relative centrifugal force, and the pellet was washed thrice in cold PBS. Crypts were seeded at a density of 200 crypts per 25 μL of Cultrex Basement Membrane Extract (BME) (3536–005–02, Bio-Techne) in Nunc^TM^ cell culture-treated 48-well plates (150687, Thermo Scientific). The BME was allowed to polymerize for 15 min at 37 °C before adding 250 μL/well of IntestiCult^TM^ organoid growth medium (06010, STEMCELL), supplemented with 100 units/mL Pen-Strep and 10 μM Y-27632 (Y0503, Sigma-Aldrich). Crypts were maintained in a 37 °C incubator with 5% CO_2_, with media changing every other day.

After organoid development, cells were maintained in human colonoid culture medium and passaged by mechanically dissociating colonoid cultures every 7 days and seeded at a 1:6 ratio, as previously described.^27^ Maintenance medium, also IFE medium, contained Advanced DMEM/F-12 (12634, Thermo Fisher), 2 mM GlutaMAX (35050061, Gibco), 10 mM HEPES (15630056, Gibco), 100 units/mL Pen-Strep, 1× B27 supplement (17504044, Gibco), 1× N2 supplement (17502048, Gibco), 0.15 nM Wnt Surrogate-Fc Fusion Protein (N001, ImmunoPrecise Antibodies), 10% R-spondin-1 conditioned medium (in house), 1% Noggin-Fc fusion protein conditioned medium (N002, ImmunoPrecise Antibodies), 50 ng/mL recombinant human EGF (AF-100–15, PeproTech), 1.25 mM *N*-acetylcysteine (A9165, Sigma-Aldrich), 10 nM Gastrin (G9145, Sigma-Aldrich), 500 nM A83–01 (2939, Bio-Techne), 100 ng/mL recombinant human IGF-1 protein (590904, BioLegend), and 50 ng/mL recombinant human FGF-2 protein (100–18B, PeproTech).

Differentiation of colonoids was initiated on day 4 postpassaging by replacing the IFE medium with IF* medium until day 11, reducing the concentration of Wnt Surrogate protein to 0.045 nM, and removing EGF. From day 7, a 48 h treatment with 10 μM Notch inhibitor DAPT (D5942, Sigma-Aldrich), 500 nM MEK inhibitor PD0325901 (Sigma-Aldrich), and 40 μM ISX-9 (4439/10, Bio-Techne) was initiated to boost differentiation. Final maturation was performed in IF* medium, and all analyses were performed on day 11.

### Generation of Human CHGA-mNeon Reporter Organoids

CRISPR-HOT technology was used to generate the CHGA-mNeon reporter organoids.^28,29^ Three plasmids—pSPgRNA (Addgene#47108), pCas9-mCherry-Frame +1 (Addgene#66940), and pCRISPaint-mNeon (Addgene#174092) containing an EF1α promoter-driven puromycin resistance gene—were transfected into human colonoids via electroporation as previously described.^30^ Electroporated cells were cultured in IFE medium and selected with 1 μg/mL puromycin after 5 days.

### Immunofluorescent Staining of Organoids

The staining of human organoids was performed as previously described.^31^ Briefly, after fixation, blocking, and permeabilization, the organoids were incubated with primary antibodies serotonin (1:200, goat; 20079, Immunostar) overnight at 4 °C. On the next day, the organoids were washed and incubated with secondary antibodies (1:500, Alexa Fluor, Invitrogen) for 1 h at room temperature. The organoids were mounted with Fluoromount-G (0100–01, Cambridge Bioscience) and air-dried in the dark. The Nikon AXR laser scanning confocal was used for imaging.

### Electrode Preparation and FSCV Measurement

The fabrication of carbon fiber microelectrodes (CFMs) and the acquisition of FSCV data were carried out as previously described.^32^ Briefly, a T-650 carbon fiber (7 μm diameter; Goodfellow) was drawn into a glass capillary (1.0 mm outer diameter, 0.58 mm inner diameter; World Precision Instruments) and pulled using a PE-22 micropipette puller (Narishige Group) to form a tight seal. The exposed fiber was manually cut to a length of 100 μm (±2 μm) and connected to a pinned stainless steel wire utilizing silver-conducting epoxy. The fiber was coated with Nafion^TM^ (Liquion Solution, LQ-1105, 5% by weight Nafion, Ion Power) by applying 1 V (*vs* Ag/AgCl) for 30 s.

Data collection was conducted using WCCV 3.06 software (Knowmad Technologies), a Dagan potentiostat (Dagan Corporation), and a Pine Research headstage (Pine Research Instrumentation). A waveform optimized for serotonin detection (0.2 to 1.0 V to −0.1 to 0.2 V *vs* Ag/AgCl at 1000 V/s) was applied. After equilibrating the electrode surface through cycling for 10 min at 60 Hz and 10 min at 10 Hz, measurements were taken at 10 Hz with 30 s per file.

### Calibrations and Stability Analysis

Calibrations and stability analysis were performed using a custom-built flow cell, as previously described.^33^ Briefly, serotonin solutions of different concentrations were injected into the flow cell for a 10 s rectangular analyte pulse. Current responses at the CFMs was correlated to serotonin concentration, and the linear concentration range was determined.

The following buffer/media compositions were tested in the flow cell: Advanced Dulbecco’s modified Eagle’s medium-Ham’s F-12 (DMEM/F-12) (12634, Thermo Fisher) supplemented with 2 mM GlutaMAX (35050061, Gibco), 10 mM HEPES (15630056, Gibco), 100 units/mL Pen–Strept, 1× B27 supplement (17504044, Gibco), and 1× N2 supplement (17502048, Gibco). Secretion buffer contains 138 mM NaCl, 4.5 mM KCl, 4.2 mM NaHCO_3_, 1.2 mM NaH_2_PO_4_, 2.6 mM CaCl_2_, 1.2 mM MgCl_2_, and 10 mM HEPES (pH 7.4) with/without 0.1% bovine serum albumin (BSA; 15260037, Gibco). The stability of the serotonin signal over 60 min was tested with repeated injections of 100 nM serotonin in the secretion buffer without BSA every 2 min.

### Colonoid Measurements

Organoids were placed in a 3.5 cm dishes of 2 mL secretion buffer-BSA on a Bio Station IM (Nikon). mNeon-reporter was used to identify ECC-containing organoid structures, and CFMs were positioned using a QUAD micromanipulator (Sutter Instruments). The setup was completed by placing the Ag/AgCl-reference electrode in the dish and adding a cover of aluminum foil connected to the ground as a Faraday cage. After cycling, 15 min of back-to-back files were taken before adding 10 μM FSK and 10 μM IBMX and recording another 15 min at the same location. For SSRI treatment, 10 μM escitalopram (escit) (Sigma-Aldrich) was added after the initial 15 min and incubated for 5 min before adding FSK/IBMX.

### Analysis

Data analysis and export of FSCV data were performed using WCCV 3.06 software (Knowmad Technologies). Graphs were generated, and statistical analysis was performed using Prism10 (GraphPad). All quantifications are shown as mean ± standard error of the mean (SEM), unless otherwise stated. Significance levels were set as follows: * *P* < 0.05, ** *P* < 0.005, and *** *P* < 0.001. Number of replicates and statistical tests are indicated in each figure legend. Schematic representations were generated using biorender.com.

## Results and Discussion

### Optimized Buffer to Facilitate Cell Functionality and FSCV Measurements

**Figure 1:**
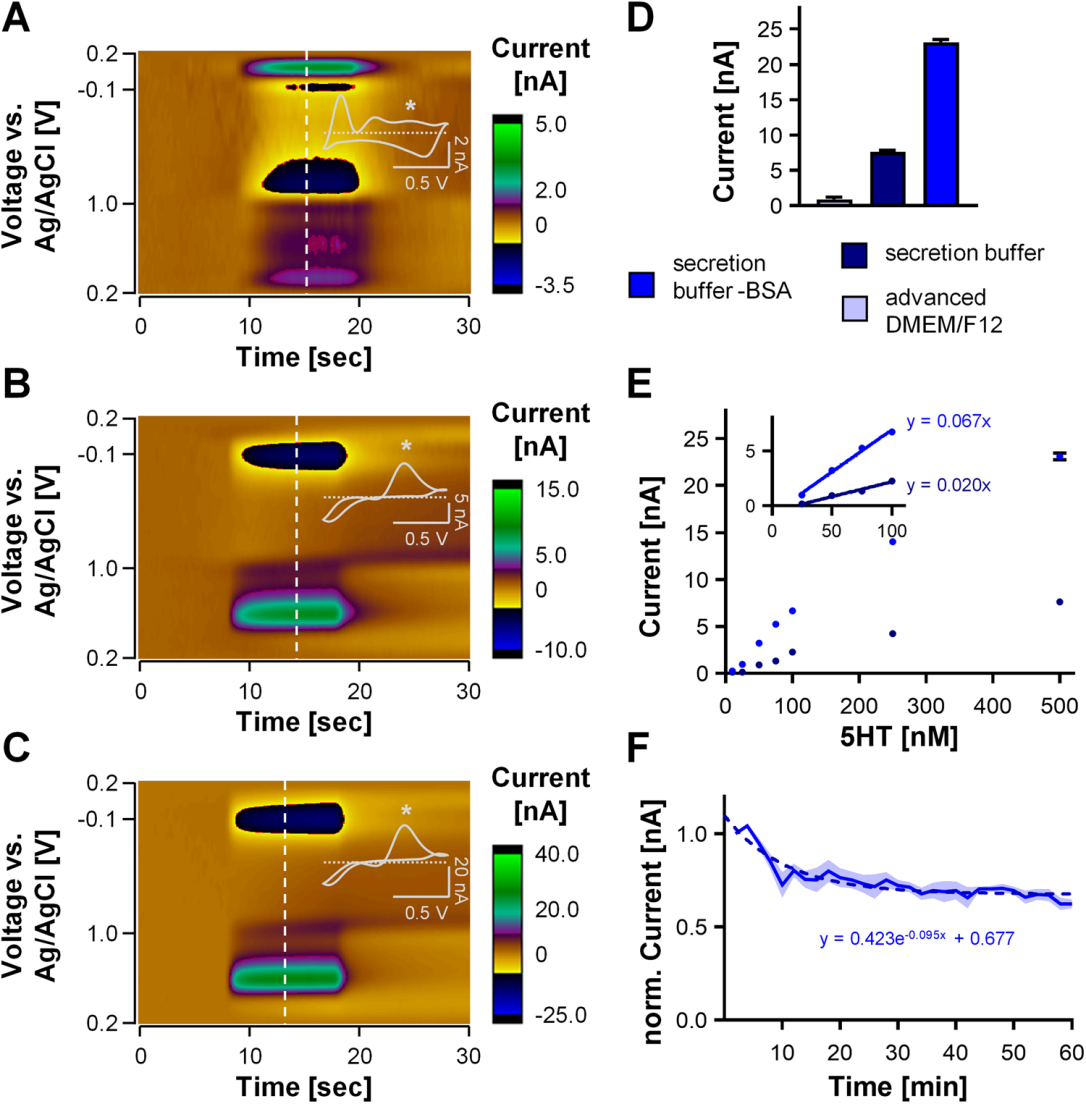
FSCV buffer optimization. (A–C) Representative color plot for 500 nM serotonin injections in advanced DMEM/F-12 (A), secretion buffer (B), and secretion buffer-BSA (C) with CV inset showing serotonin-specific oxidation peak (*). (D) Peak current measured from 500 nM serotonin injections in the different buffers/media (*n* = 9 measurements on 3 electrodes, mean ± SEM). (E) Calibration curve for secretion buffer with and without BSA showing serotonin concentration *vs* peak current and regression of the linear range in the inset (*n* = 3 for each buffer, mean ± SEM). (F) Stability analysis for 100 nM serotonin injections in secretion buffer-BSA over a total of 60 min with exponential decay fitting as dashed line (*n* = 4, mean ± SEM).

When optimizing electroanalytical measurements in novel biological systems, it is important to characterize the electrochemical response in the cell media specific for the cell type being studied. This is because these media contain a complex variety of substrates and proteins that could interact negatively with the sensors. For example, in previous work, we found that we could not use a common culture medium for neurons, Neurobasal medium, to measure serotonin from human-derived serotonergic neurons with FSCV.^32^ Exposure of this medium to the electrode dramatically decreased the analytical response to serotonin. In this previous work, we found that we could keep the cells alive for the duration of the experiment in HEPES buffer that included glucose.^32^ Here, we performed a similar experiment where we utilized advanced DMEM/F-12, which is the colonoid culture medium, as the flow injection buffer during an FSCV injection of serotonin (500 nM). Figure 1A shows the FSCV color plot of this injection. Interpretation of these color plots can be aided via reference to Michael et al.^34^ Briefly, voltage is plotted on the *y* axis with time on the *x* axis and current in false color. The absence of a stereotypical CV response to serotonin shows that this medium, as with the Neurobasal medium, has reduced the capacity of the electrode to respond to serotonin. This is likely because of fouling of the surface by amino acids in the medium that have the capacity to electropolymerize on the electrode surface.^32,35−37^ We quantified the peak at approximately 0.6 V (where we expect to capture serotonin oxidation) and found 0.92 ± 0.27 nA. When we repeated the experiment in the secretion buffer used routinely for secretion analysis of gut organoids by mass spectroscopy,^38^ we captured a typical serotonin CV, and a much higher signal of 7.63 ± 0.23 nA (Figure 1B).

Secretion buffer contains 0.1% BSA, which prevents the attachment of organoids and analytes to plastic surfaces. We find that the presence of BSA reduces the FSCV serotonin signal, again, likely because of fouling of the electrode by BSA’s electroactive amino acid residues. Auspiciously, for this type of real-time, end-point FSCV experiments, this attachment is not an important consideration; thus, BSA can be excluded from the buffer. When removing the BSA, the serotonin signal was 23.11 ± 0.39 nA (Figure 1C), which is comparable to signals we have seen before in TRIS buffer.^33^Figure 1D shows the pooled responses of the three buffers, and Figure 1E compares calibration curves between the secretion buffer with and without BSA. Calibrations show that without the BSA, the response of the electrode is more sensitive, with linear regression slopes of 0.076 ± 0.003 nA nM^–1^ (secretion buffer-BSA) and 0.027 ± 0.001 nA nM^–1^ (secretion buffer).

Given the superior response in BSA-free buffer, we assessed the stability of the response to repeated injections of serotonin and found that the electrode reached stability in a profile that could be fit with an exponential decay over 60 min (Figure 1F), which is consistent with the stability of the serotonin signal in TRIS buffer.^33^ Therefore, having established the suitability of the secretion buffer minus BSA for FSCV experiment, we proceeded with our physiological experiments using this buffer.

### Characterization of the Colonoids Reveals the Presence of Serotonin-Producing ECCs

**Figure 2:**
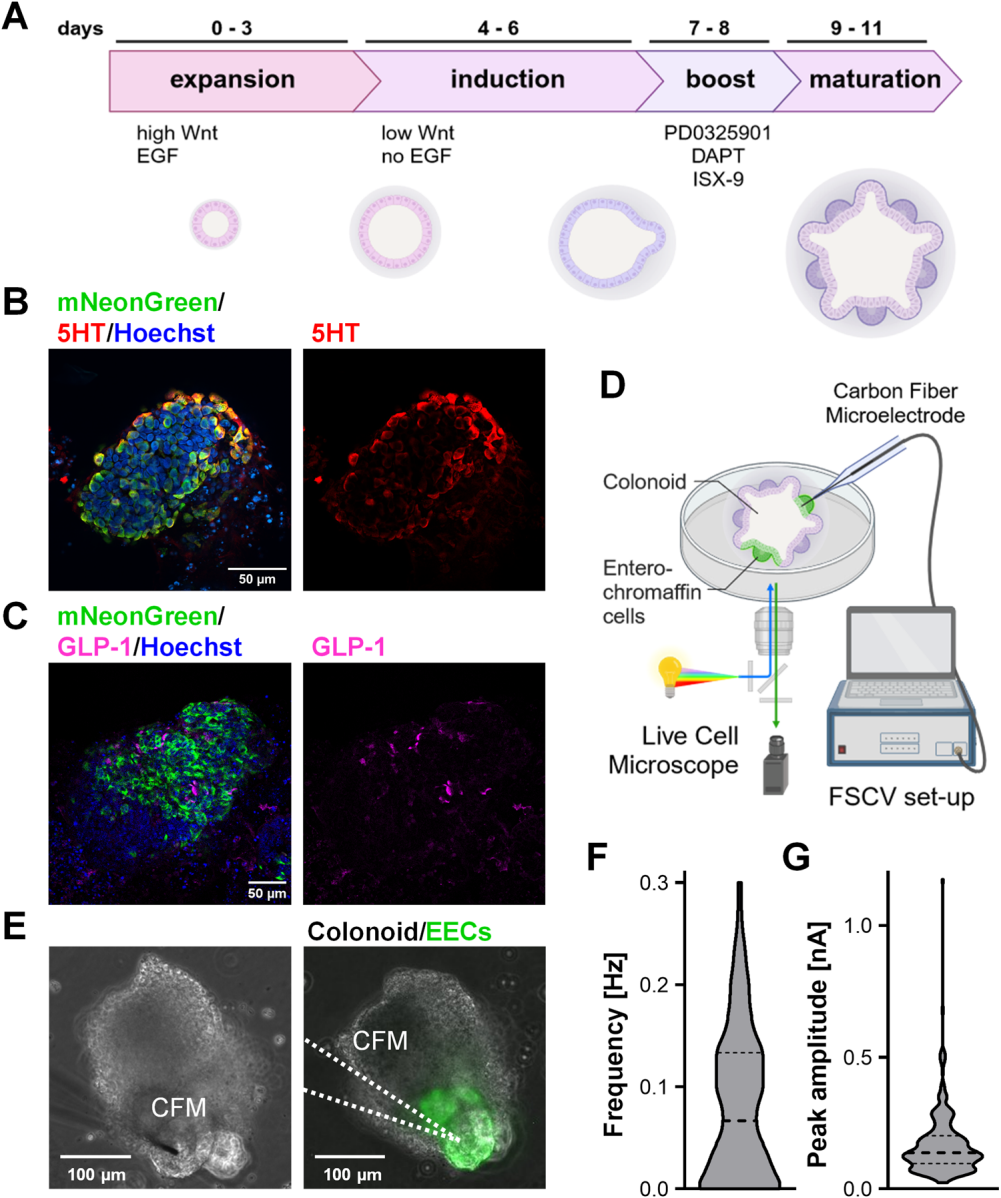
Characterization of serotonin-secreting cells in human colonoids by immunofluorescence and FSCV. (A) Schematic showing the differentiation protocol of human colonoids for the enrichment of enteroendocrine cells. (B, C) Whole-mount immunostaining of fixed CHGA-mNeonGreen human colonoids stained for ECC synthesizing serotonin (B) and enteroendocrine marker GLP-1 (C) with Hoechst nuclear counterstain (blue). Scale bar: 50 μm. (D) Scheme of FSCV setup for colonoid measurements. (E) Dish containing colonoids is placed on a live-cell microscope to identify mNeonGreen-expressing structures and provide temperature control. Scale bar: 100 μm. (F) Frequency of spontaneous serotonin release events (*n* = 90 files from 3 organoids, violin plot with median and quantiles as dashed lines). (G) Peak amplitudes of serotonin release (*n* = 220 events from 3 organoids, violin plot with median and quantiles as dashed lines). Schemes in panels (A, D) were created by biorender.com.

A previous work using fluorescent reporters has greatly contributed to our knowledge of ECC distribution, differentiation, and function but is derived from murine models.^24,25,39^ Therefore, as an *in vitro* model system for the human gut, we derived human colon organoids (colonoids) from primary colonic crypts. The expansion and differentiation protocol, designed to foster ECC development (Figure 2A), is described in detail elsewhere.^40^ To identify endocrine structures, we used the expression of our recently developed mNeonGreen reporter under the control of the CHGA promoter. Immunostainings confirmed the enrichment of ECCs synthesizing serotonin in mNeonGreen-positive structures (Figure 2B), while a smaller fraction of mNeonGreen-positive cells express glucagon-like peptide 1 (GLP-1) as a marker for enteroendocrine cells (Figure 2C). Therefore, the mNeonGreen reporter represents a valuable tool to identify serotonin-producing ECCs in living colonoids.

Chemical measurements from human-derived models are an emerging research tool, providing invaluable insights into the functionality of the derived somatic cells. Measurement of neurotransmitter production from iPSC-derived neurons has been reported for dopaminergic^41^ as well as serotonergic neurons.^32,42^ A first study employing differential pulse voltammetry was used to study the variability in the development of human kidney organoids.^43^ We immersed the colonoids in BSA-free secretion buffer in a dish on a live-cell microscope (Figure 2D). We identified the ECCs via the expression of the mNeonGreen reporter and placed the electrode on the surface of the ECC-containing structures (Figure 2E). After 20 min of equilibration time, we took FSCV files for 15 min. We observed sparse but robust spontaneous electrochemical events during this control time, with a mean frequency of 0.082 ± 0.00 Hz and a mean amplitude of 0.169 ± 0.00 nA (Figure 2F,G). The frequency of these events mirrors those seen previously in stimulated BON cells.^19^

We provide here evidence of spontaneous secretion from the human-derived organoids. The CVs of these events strongly resemble FSCV serotonin;^32,44^ therefore, we next performed some pharmacology to validate the identity.

### Spontaneous Release Events Increase their Frequency and Amplitude with cAMP Agonism

**Figure 3:**
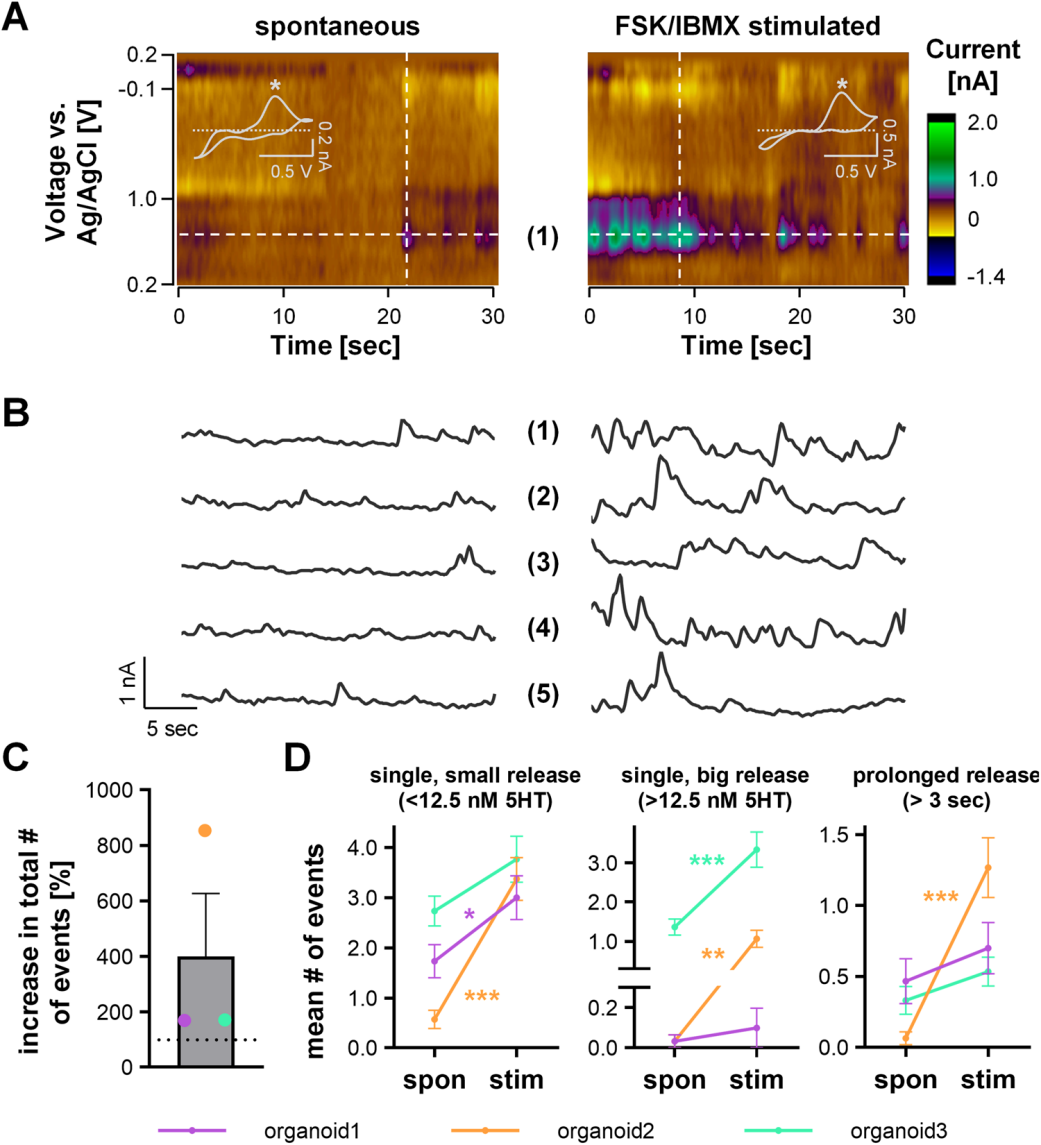
FSCV analysis of spontaneous and stimulated serotonin release from colonoids. (A) Representative color plots with CV inset showing the serotonin-specific oxidation peak (*) and (B) IT curves of spontaneous and FSK/IBMX-stimulated release (10 μM FSK and 10 μM IBMX). (C) Percent increase of total number of serotonin release events over 15 min after stimulation (*n* = 3 organoids, mean ± SEM). (D) Mean number of small (<12.5 nM serotonin), big (>12.5 nM serotonin), and prolonged (>3 s) release events per file (*n* = 30 files per organoid; mean ± SEM; two-way ANOVA with Sidak’s post hoc test, * *p* < 0.05, ** *p* < 0.01, and *** *p* < 0.001).

A variety of stimuli have been employed to release serotonin from previous gut models, including ionomycin, a Ca^2+^ ionophore,^20^ high extracellular Ca^2+^^19^ and mechanical stimulation.^18^ In this work, we stimulated these colonoids with an adenylate cyclase activator, FSK, and a phosphodiesterase inhibitor, IBMX, both of which increase intracellular cAMP. cAMP increases the secretory capacity from rat biliary epithelium^45^ and FSK potentiates insulin secretion from rat islets.^46^ The results of this stimulation are shown in Figure 3. Representative color plots and IT curves are shown in Figure 3A,B, left panel, with a signature serotonin CV. FSK and IBMX increase the intracellular levels of the second messenger cAMP and activate protein-kinase, and in turn, are thought to induce serotonin release by exocytosis. The combination of those two agents has been previously described to induce GLP-1 release from enteroendocrine cells via intracellular Ca^2+^ signaling.^47^ In line with this, we found a clear increase in the total number of events (by 399.1 ± 394.8%), however, with quite some organoid-to-organoid variation (*n* = 3 organoids, Figure 3C). Similarly, the mean number of release events per file increased for each organoid, with significant increases in small and big events below and above 12.5 nM of serotonin released in 2 organoids each and an increase in prolonged releases longer than 3 s in one of the organoids (Figure 3D). We believe these variations are dependent on the position of the electrode with respect to the density of ECCs within the colonoid structure and the size of the structure. This experiment has demonstrated that colonoids increase their secretion activity when stimulated with FSK/IBMX.

### Real-Time Serotonin Signaling from Colonoids is Modulated by Serotonin Transporter Activity

**Figure 4:**
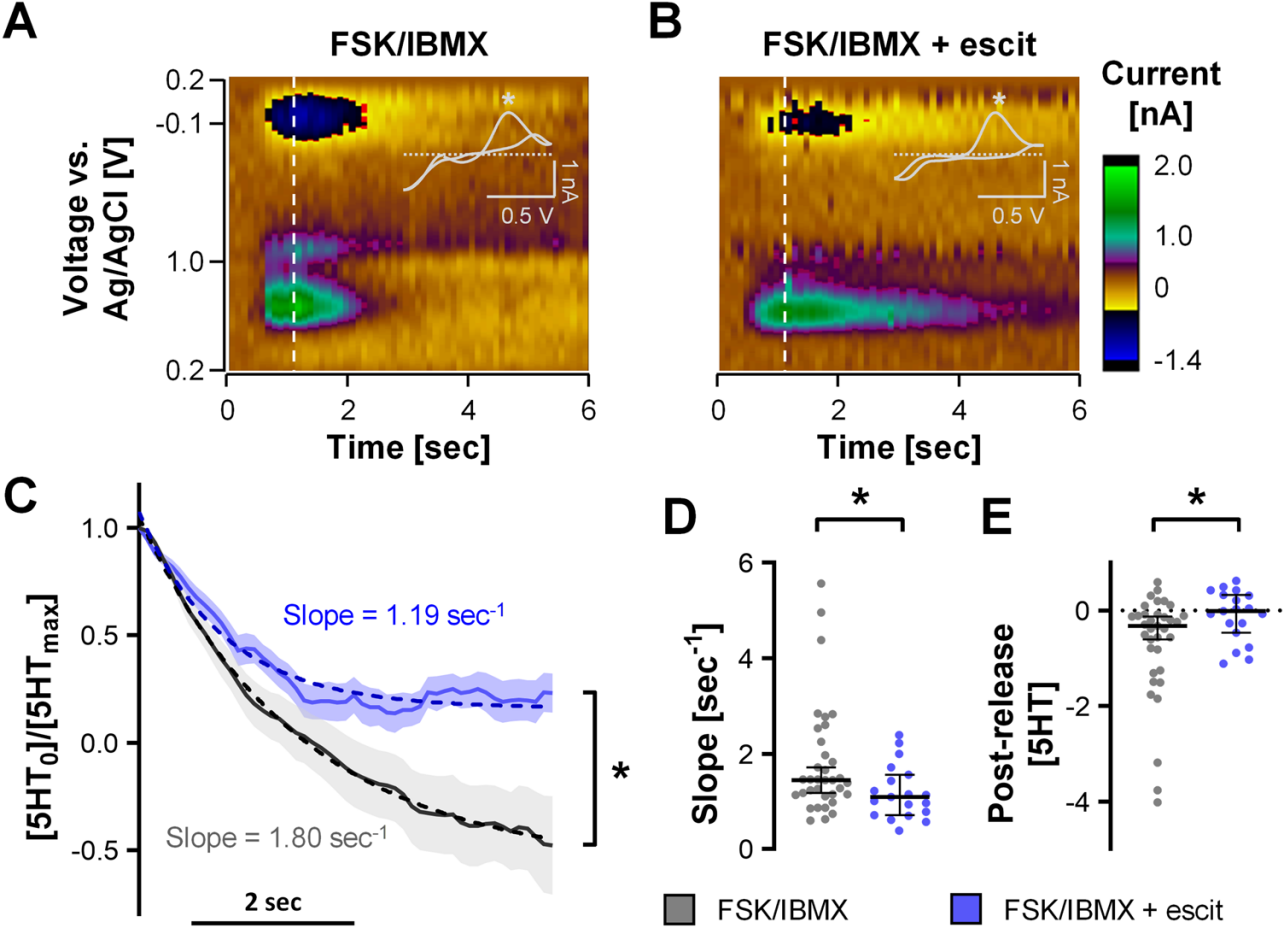
Escitalopram treatment blocks serotonin reuptake in the gut endothelium. (A, B) Representative color plots with CV inset showing serotonin-specific oxidation peak (*) under FSK/IBMX stimulation condition without (A) and with pretreatment with 10 uM escit (B). (C) Normalized serotonin concentration starting from the peak of release events with (blue) and without (gray) pretreatment with escit and exponential decay fitting as dashed lines (FSK/IBMX: *n* = 35; FSK/IMBX+escit = 19 events from 3 organoids, mean ± SEM; two-way ANOVA with Sidak’s post hoc test, * *p* < 0.05). (D, E) Slopes and postrelease serotonin concentration calculated from exponential decay fitting of individual release events (FSK/IBMX: *n* = 33; FSK/IMBX+escit = 19 events from 3 organoids, median with 95% confidence interval; unpaired *t* test, * *p* < 0.05).

To further validate the chemical identity of the signal, we employed a SERT inhibiting agent. SERTs are transmembrane transporters that reuptake serotonin with high capacity and are inhibited by the popular antidepressants, the SSRIs.^48,49^ We and others have found in the brain that SERT inhibition with SSRI exerts profound effects on the FSCV serotonin signal.^50−54^ SERTs are not only found in the brain but are also highly concentrated in the gut.^55^ Indeed, SSRIs have clinical utility for treating IBS.^56^ Therefore, we employed escit to test the effect on the FSCV signal in the organoids. Figure 4A is a color plot, representative of an isolated FSK/IBMX induced release, and Figure 4B is a representative plot of a similar event 5 min after treatment with escit. Visually, the duration of the signal appears longer after SSRI and to verify this notion, we employed exponential fitting of the reuptake curves across 35 and 19 events for control and escit, respectively (Figure 4C). We found a significant decrease in the initial slope (FSK/IBMX = 1.80 ± 0.20 s^–1^, FSK/IBMX + escit = 1.19 ± 0.13 s^–1^) indicating the inhibition of reuptake by SERTs (Figure 4C,D). Interestingly, there was a significant increase in the mean steady state serotonin concentration postevent by 0.7337([5HT_0_/5HT_max_]) (Figure 4C,E). We have previously observed this effect before *in vivo* in the mouse brain, which we attributed to SSRI-induced SERT internalization.^51,57^ Based on this finding, an interesting future area of study would be to investigate whether SERT internalization applies to gut epithelial cells.

Our study resembles a previous work by Yeoman et al., who found that SSRIs decreased the slope of their amperometric signal in colonic tissues.^21^ As above, SSRIs are clinically prescribed to treat IBS.^56^ The clinical use of antidepressants for IBS is based on 3 theories.^58^ The first is that because IBS and depression are highly comorbid,^6^ the therapeutic effects of SSRIs on depression might, in turn, reduce the severity of IBS. The second is based on the central analgesic actions of antidepressants and their effects in restoring serotonin homeostasis.^59,60^ Finally, it has previously been postulated that these agents may interact locally with the gut and the intestinal barrier.^61−63^ While we cannot localize the precise mechanism with our study of the three theories, we provide compelling evidence for the direct action of SSRIs on gut serotonin.

## Conclusions

The gut is increasingly recognized to play a central role in modulating the brain–body immune axis. The gut and the brain share many signaling molecules, in particular serotonin. Stem cell-derived, human models open interesting avenues for studying human-specific phenomena in well-controlled, *in vitro* environments; however, models such as these have not widely been probed with electrochemistry. Here, we described FSCV measurements of serotonin signaling in newly generated CHGA reporter organoids, where primary human ECCs are fluorescently labeled for easy identification.

We first biochemically characterized the gut organoids and found that they contained the essential cell type producing and releasing serotonin in the gut, ECCs. Then, we optimized an *in vitro* buffer for cell culture and FSCV measurements and measured spontaneous events that increased in frequency and amplitude upon stimulation with FSK/IMBX. Finally, the identity of the signal was validated to be serotonin with an SSRI, which significantly slowed the reuptake profile of the signal. Therefore, we presented here the first FSCV measurements in an organoid model and also a new model for measuring serotonin transmission in a human-derived gut system that we believe will be invaluable for future studies of the roles of serotonin in gut health and disease.

## References

[ref1] Sikander A.; Rana S. V.; Prasad K. K. Role of serotonin in gastrointestinal motility and irritable bowel syndrome. Clin. Chim. Acta 2009, 403 (1–2), 47–55. 10.1016/j.cca.2009.01.028. 19361459

[ref2] Crowell M. D. Role of serotonin in the pathophysiology of the irritable bowel syndrome. Br. J. Pharmacol. 2004, 141 (8), 1285–1293. 10.1038/sj.bjp.0705762. 15100164 PMC1574906

[ref3] Vahora I. S.; Tsouklidis N.; Kumar R.; Soni R.; Khan S. How Serotonin Level Fluctuation Affects the Effectiveness of Treatment in Irritable Bowel Syndrome. Cureus 2020, 12 (9), e9871 10.7759/cureus.9871. 32968548 PMC7505258

[ref4] Cui L.; Li S.; Wang S.; Wu X.; Liu Y.; Yu W.; Wang Y.; Tang Y.; Xia M.; Li B. Major depressive disorder: hypothesis, mechanism, prevention and treatment. Signal Transduction Target Ther. 2024, 9 (1), 30 10.1038/s41392-024-01738-y. PMC10853571 38331979

[ref5] Coppen A. The Biochemistry of Affective Disorders. Br J. Psychiatry 1967, 113 (504), 1237–1264. 10.1192/bjp.113.504.1237. 4169954

[ref6] Staudacher H. M.; Black C. J.; Teasdale S. B.; Mikocka-Walus A.; Keefer L. Irritable bowel syndrome and mental health comorbidity–approach to multidisciplinary management. Nat. Rev. Gastroenterol. Hepatol. 2023, 20 (9), 582–596. 10.1038/s41575-023-00794-z. 37268741 PMC10237074

[ref7] El-Merahbi R.; Löffler M.; Mayer A.; Sumara G. The roles of peripheral serotonin in metabolic homeostasis. FEBS Lett. 2015, 589 (15), 1728–1734. 10.1016/j.febslet.2015.05.054. 26070423

[ref8] Martin A. M.; Lumsden A. L.; Young R. L.; Jessup C. F.; Spencer N. J.; Keating D. J. Regional differences in nutrient-induced secretion of gut serotonin. Physiol. Rep. 2017, 5 (6), e13199 10.14814/phy2.13199. 28320893 PMC5371566

[ref9] Kendig D. M.; Grider J. R. Serotonin and colonic motility. Neurogastroenterol. Motil. 2015, 27 (7), 899–905. 10.1111/nmo.12617. 26095115 PMC4477275

[ref10] Hansen M. B.; Witte A. The role of serotonin in intestinal luminal sensing and secretion. Acta Physiol. 2008, 193 (4), 311–323. 10.1111/j.1748-1716.2008.01870.x. 18462271

[ref11] Perez F.; Kotecha N. A.; Lavoie B.; Mawe G. M.; Patel B. A. Monitoring Gut Epithelium Serotonin and Melatonin Overflow Provides Spatial Mapping of Inflammation. ChemBioChem 2022, 24 (2), e202200334 10.1002/cbic.202200334. 36394122 PMC9909162

[ref12] Nzakizwanayo J.; Dedi C.; Standen G.; Macfarlane W. M.; Patel B. A.; Jones B. V. *Escherichia coli* Nissle 1917 enhances bioavailability of serotonin in gut tissues through modulation of synthesis and clearance. Sci. Rep. 2015, 5, 17324 10.1038/srep17324. 26616662 PMC4663480

[ref13] Khlevner J.; Park Y.; Margolis K. G. Brain–Gut Axis. Gastroenterol. Clin. North Am. 2018, 47 (4), 727–739. 10.1016/j.gtc.2018.07.002. 30337029 PMC6829582

[ref14] Chin A.; Svejda B.; Gustafsson B. I.; Granlund A. B.; Sandvik A. K.; Timberlake A.; Sumpio B.; Pfragner R.; Modlin I. M.; Kidd M. The role of mechanical forces and adenosine in the regulation of intestinal enterochromaffin cell serotonin secretion. Am. J. Physiol.: Gastrointest. Liver Physiol. 2012, 302 (3), G397–G405. 10.1152/ajpgi.00087.2011. 22038827 PMC3287403

[ref15] Keszthelyi D.; Troost F. J.; Jonkers D. M.; Van Eijk H. M.; Lindsey P. J.; Dekker J.; Buurman W. A.; Masclee A. A. M. Serotonergic reinforcement of intestinal barrier function is impaired in irritable bowel syndrome. Aliment. Pharmacol. Ther. 2014, 40 (4), 392–402. 10.1111/apt.12842. 24943480

[ref16] Fung T. C.; Olson C. A.; Hsiao E. Y. Interactions between the microbiota, immune and nervous systems in health and disease. Nat. Neurosci. 2017, 20 (2), 145–155. 10.1038/nn.4476. 28092661 PMC6960010

[ref17] Wu H.; Denna T. H.; Storkersen J. N.; Gerriets V. A. Beyond a neurotransmitter: The role of serotonin in inflammation and immunity. Pharmacol. Res. 2019, 140, 100–114. 10.1016/j.phrs.2018.06.015. 29953943

[ref18] Patel B. A. Continuous amperometric detection of co-released serotonin and melatonin from the mucosa in the ileum. Analyst 2008, 133 (4), 516–524. 10.1039/b717034c. 18365122

[ref19] Wang Y.; Gu C.; Patel B. A.; Ewing A. G. Nano-analysis Reveals High Fraction of Serotonin Release during Exocytosis from a Gut Epithelium Model Cell. Angew. Chem., Int. Ed. 2021, 60 (44), 23552–23556. 10.1002/anie.202108193. PMC8597005 34363735

[ref20] Shaaban A.; Maaß F.; Schwarze V.; Lund M. L.; Beuermann S.; Chan M.; Harenberg C.; Bewick G. A.; Keating D. J.; Benseler F.; Cooper B. H.; Imig C. Dissecting Functional, Structural, and Molecular Requirements for Serotonin Release from Mouse Enterochromaffin Cells bioRxiv 2021.

[ref21] Yeoman M. S.; Fidalgo S.; Marcelli G.; Patel B. A. Amperometry approach curve profiling to understand the regulatory mechanisms governing the concentration of intestinal extracellular serotonin. Sci. Rep. 2024, 14 (1), 10479 10.1038/s41598-024-61296-9. 38714793 PMC11076564

[ref22] Li J.; Liu Y.; Yuan L.; Zhang B.; Bishop E. S.; Wang K.; Tang J.; Zheng Y.; Xu W.; Niu S.; Beker L.; Li T. L.; Chen G.; Diyaolu M.; Thomas A.; Mottini V.; Tok N. H.; Dunn J. C. Y.; Cui B.; Paşca S. P.; Cui Y.; Habtezion A.; Chen X.; Bao Z. A tissue-like neurotransmitter sensor for the brain and gut. Nature 2022, 606 (7912), 94–101. 10.1038/s41586-022-04615-2. 35650358 PMC9210986

[ref23] Delong L. M.; Witt C. E.; Pennell M.; Ross A. E. A microfluidic chip for sustained oxygen gradient formation in the intestine ex vivo. Lab Chip 2024, 24 (7), 1918–1929. 10.1039/D3LC00793F. 38372633 PMC10998727

[ref24] Billing L. J.; Larraufie P.; Lewis J.; Leiter A.; Li J.; Lam B.; Yeo G. S.; Goldspink D. A.; Kay R. G.; Gribble F. M.; Reimann F. Single cell transcriptomic profiling of large intestinal enteroendocrine cells in mice – Identification of selective stimuli for insulin-like peptide-5 and glucagon-like peptide-1 co-expressing cells. Mol. Metab. 2019, 29, 158–169. 10.1016/j.molmet.2019.09.001. 31668387 PMC6812004

[ref25] Song Y.; Fothergill L. J.; Lee K. S.; Liu B. Y.; Koo A.; Perelis M.; Diwakarla S.; Callaghan B.; Huang J.; Wykosky J.; Furness J. B.; Yeo G. W. Stratification of enterochromaffin cells by single-cell expression analysis bioRxiv 2023. 10.7554/eLife.90596 PMC11970908 40184163

[ref26] Bellono N. W.; Bayrer J. R.; Leitch D. B.; Castro J.; Zhang C.; O’donnell T. A.; Brierley S. M.; Ingraham H. A.; Julius D. Enterochromaffin Cells Are Gut Chemosensors that Couple to Sensory Neural Pathways. Cell 2017, 170 (1), 185–198.e16. 10.1016/j.cell.2017.05.034. 28648659 PMC5839326

[ref27] Goldspink D. A.; Lu V. B.; Miedzybrodzka E. L.; Smith C. A.; Foreman R. E.; Billing L. J.; Kay R. G.; Reimann F.; Gribble F. M. Labeling and Characterization of Human GLP-1-Secreting L-cells in Primary Ileal Organoid Culture. Cell Rep. 2020, 31 (13), 107833 10.1016/j.celrep.2020.107833. 32610134 PMC7342002

[ref28] Beumer J.; Puschhof J.; Bauzá-Martinez J.; Martínez-Silgado A.; Elmentaite R.; James K. R.; Ross A.; Hendriks D.; Artegiani B.; Busslinger G. A.; Ponsioen B.; Andersson-Rolf A.; Saftien A.; Boot C.; Kretzschmar K.; Geurts M. H.; Bar-Ephraim Y. E.; Pleguezuelos-Manzano C.; Post Y.; Begthel H.; Van Der Linden F.; Lopez-Iglesias C.; Van De Wetering W. J.; Van Der Linden R.; Peters P. J.; Heck A. J. R.; Goedhart J.; Snippert H.; Zilbauer M.; Teichmann S. A.; Wu W.; Clevers H. High-Resolution mRNA and Secretome Atlas of Human Enteroendocrine Cells. Cell 2020, 181 (6), 1291–1306.e19. 10.1016/j.cell.2020.04.036. 32407674

[ref29] Artegiani B.; Hendriks D.; Beumer J.; Kok R.; Zheng X.; Joore I.; De Sousa Lopes S. C.; Van Zon J.; Tans S.; Clevers H. Fast and efficient generation of knock-in human organoids using homology-independent CRISPR–Cas9 precision genome editing. Nat. Cell Biol. 2020, 22 (3), 321–331. 10.1038/s41556-020-0472-5. 32123335

[ref30] Gaebler A.-M.; Hennig A.; Buczolich K.; Weitz J.; Welsch T.; Stange D. E.; Pape K. Universal and Efficient Electroporation Protocol for Genetic Engineering of Gastrointestinal Organoids. J. Visualized Exp. 2020, (156), e60704 10.3791/60704-v. 32150173

[ref31] Tsakmaki A.; Pedro P. F.; Pavlidis P.; Hayee B.; Bewick G. A. ISX-9 manipulates endocrine progenitor fate revealing conserved intestinal lineages in mouse and human organoids. Mol. Metab. 2020, 34, 157–173. 10.1016/j.molmet.2020.01.012. 32180555 PMC7036449

[ref32] Holmes J.; Lau T.; Saylor R.; Fernández-Novel N.; Hersey M.; Keen D.; Hampel L.; Horschitz S.; Ladewig J.; Parke B.; Reed M. C.; Nijhout H. F.; Best J.; Koch P.; Hashemi P. Voltammetric Approach for Characterizing the Biophysical and Chemical Functionality of Human Induced Pluripotent Stem Cell-Derived Serotonin Neurons. Anal. Chem. 2022, 94 (25), 8847–8856. 10.1021/acs.analchem.1c05082. 35713335

[ref33] Hexter M.; Van Batenburg-Sherwood J.; Hashemi P. Novel Experimental and Analysis Strategies for Fast Voltammetry: 2. A Troubleshoot-Free Flow Cell for FSCV Calibrations. ACS Meas. Sci. Au 2023, 3 (2), 120–126. 10.1021/acsmeasuresciau.2c00059. 37090258 PMC10120031

[ref34] Michael D. J.; Joseph J. D.; Kilpatrick M. R.; Travis E. R.; Wightman R. M. Improving Data Acquisition for Fast-Scan Cyclic Voltammetry. Anal. Chem. 1999, 71 (18), 3941–3947. 10.1021/ac990491+. 10500480

[ref35] Jang J.; Cho N. U.; Hwang S.; Kwak Y.; Kwon H.; Heien M. L.; Bennet K. E.; Oh Y.; Shin H.; Lee K. H.; Jang D. P. Understanding the different effects of fouling mechanisms on working and reference electrodes in fast-scan cyclic voltammetry for neurotransmitter detection. Analyst 2024, 149 (10), 3008–3016. 10.1039/D3AN02205F. 38606455 PMC11648937

[ref36] Takmakov P.; Zachek M. K.; Keithley R. B.; Walsh P. L.; Donley C.; Mccarty G. S.; Wightman R. M. Carbon Microelectrodes with a Renewable Surface. Anal. Chem. 2010, 82 (5), 2020–2028. 10.1021/ac902753x. 20146453 PMC2838506

[ref37] Shin D.; Tryk D. A.; Fujishima A.; Merkoçi A.; Wang J. Resistance to Surfactant and Protein Fouling Effects at Conducting Diamond Electrodes. Electroanalysis 2005, 17, 305–311. 10.1002/elan.200403104.

[ref38] Miedzybrodzka E. L.; Foreman R. E.; Galvin S. G.; Larraufie P.; George A. L.; Goldspink D. A.; Reimann F.; Gribble F. M.; Kay R. G. Organoid Sample Preparation and Extraction for LC-MS Peptidomics. STAR Protoc 2020, 1 (3), 100164 10.1016/j.xpro.2020.100164. 33377058 PMC7757358

[ref39] Shajib M. S.; Wang H.; Kim J. J.; Sunjic I.; Ghia J.; Denou E.; Collins M.; Denburg J. A.; Khan W. I. Interleukin 13 and Serotonin: Linking the Immune and Endocrine Systems in Murine Models of Intestinal Inflammation. PLoS One 2013, 8 (8), e72774 10.1371/journal.pone.0072774. 24015275 PMC3755966

[ref40] Lei Y.; Bohl B.; Meyer L.; Jacobs M.; Haq N.; Yang X.; Bu’ Hussain H.; Hayee; Murphy K. G.; Hashemi P.; Bewick G. A. Mapping the druggable targets displayed by human colonic enteroendocrine cells bioRxiv 2024.

[ref41] Zanetti C.; Spitz S.; Berger E.; Bolognin S.; Smits L. M.; Crepaz P.; Rothbauer M.; Rosser J. M.; Marchetti-Deschmann M.; Schwamborn J. C.; Ertl P. Monitoring the neurotransmitter release of human midbrain organoids using a redox cycling microsensor as a novel tool for personalized Parkinson’s disease modelling and drug screening. Analyst 2021, 146 (7), 2358–2367. 10.1039/D0AN02206C. 33625407

[ref42] Nakatsuka N.; Heard K. J.; Faillétaz A.; Momotenko D.; Vörös J.; Gage F. H.; Vadodaria K. C. Sensing serotonin secreted from human serotonergic neurons using aptamer-modified nanopipettes. Mol. Psychiatry 2021, 26 (7), 2753–2763. 10.1038/s41380-021-01066-5. 33767349 PMC9997689

[ref43] Suhito I. R.; Kim J. W.; Koo K.; Nam S. A.; Kim Y. K.; Kim T. In Situ Detection of Kidney Organoid Generation From Stem Cells Using a Simple Electrochemical Method. Adv. Sci. 2022, 9 (20), e2200074 10.1002/advs.202200074. PMC9284177 35506260

[ref44] Wood K. M.; Zeqja A.; Nijhout H. F.; Reed M. C.; Best J.; Hashemi P. Voltammetric and mathematical evidence for dual transport mediation of serotonin clearance in vivo. J. Neurochem. 2014, 130 (3), 351–359. 10.1111/jnc.12733. 24702305 PMC4107184

[ref45] Ammon H. P. T.; Müller A. B. Effect of forskolin on islet cyclic AMP, insulin secretion, blood glucose and intravenous glucose tolerance in rats. Naunyn-Schmiedeberg’s Arch. Pharmacol. 1984, 326 (4), 364–367. 10.1007/BF00501444. 6090960

[ref46] Francis H.; Glaser S.; Ueno Y.; Lesage G.; Marucci L.; Benedetti A.; Taffetani S.; Marzioni M.; Alvaro D.; Venter J.; Reichenbach R.; Fava G.; Phinizy J. L.; Alpini G. cAMP stimulates the secretory and proliferative capacity of the rat intrahepatic biliary epithelium through changes in the PKA/Src/MEK/ERK1/2 pathway. J. Hepatol. 2004, 41 (4), 528–537. 10.1016/j.jhep.2004.06.009. 15464232

[ref47] Simpson A. K.; Ward P. S.; Wong K. Y.; Collord G. J.; Habib A. M.; Reimann F.; Gribble F. M. Cyclic AMP triggers glucagon-like peptide-1 secretion from the GLUTag enteroendocrine cell line. Diabetologia 2007, 50 (10), 2181–2189. 10.1007/s00125-007-0750-9. 17643200 PMC7212076

[ref48] Blackburn K. J.; French P. C.; Merrills R. J. 5-hydroxytryptamine uptake by rat brain in vitro. Life Sci. 1967, 6 (15), 1653–1663. 10.1016/0024-3205(67)90176-2. 5299290

[ref49] Coleman J. A.; Green E. M.; Gouaux E. X-ray structures and mechanism of the human serotonin transporter. Nature 2016, 532 (7599), 334–339. 10.1038/nature17629. 27049939 PMC4898786

[ref50] Dunham K. E.; Venton B. J. SSRI antidepressants differentially modulate serotonin reuptake and release in Drosophila. J. Neurochem. 2022, 162 (5), 404–416. 10.1111/jnc.15658. 35736504 PMC9427694

[ref51] Witt C. E.; Mena S.; Holmes J.; Hersey M.; Buchanan A. M.; Parke B.; Saylor R.; Honan L. E.; Berger S. N.; Lumbreras S.; Nijhout F. H.; Reed M. C.; Best J.; Fadel J.; Schloss P.; Lau T.; Hashemi P. Serotonin is a common thread linking different classes of antidepressants. Cell Chem. Biol. 2023, 30 (12), 1557–1570.e6. 10.1016/j.chembiol.2023.10.009. 37992715

[ref52] Mena S.; Cruikshank A.; Best J.; Nijhout H. F.; Reed M. C.; Hashemi P. Modulation of serotonin transporter expression by escitalopram under inflammation. Commun. Biol. 2024, 7 (1), 710 10.1038/s42003-024-06240-3. 38851804 PMC11162477

[ref53] Wood K. M.; Hashemi P. Fast-Scan Cyclic Voltammetry Analysis of Dynamic Serotonin Reponses to Acute Escitalopram. ACS Chem. Neurosci. 2013, 4 (5), 715–720. 10.1021/cn4000378. 23597074 PMC3656741

[ref54] Dankoski E. C.; Carroll S.; Wightman R. M. Acute selective serotonin reuptake inhibitors regulate the dorsal raphe nucleus causing amplification of terminal serotonin release. J. Neurochem. 2016, 136 (6), 1131–1141. 10.1111/jnc.13528. 26749030 PMC4939133

[ref55] Gill R. K.; Pant N.; Saksena S.; Singla A.; Nazir T. M.; Vohwinkel L.; Turner J. R.; Goldstein J.; Alrefai W. A.; Dudeja P. K. Function, expression, and characterization of the serotonin transporter in the native human intestine. Am. J. Physiol. Gastrointest. Liver Physiol. 2008, 294 (1), G254–G262. 10.1152/ajpgi.00354.2007. 17991706 PMC4880408

[ref56] Fritsch P.; Kolber M. R.; Korownyk C. Antidepressants for irritable bowel syndrome. Can. Fam Physician 2020, 66 (4), 265. 32273413 PMC7145118

[ref57] Lau T.; Horschitz S.; Berger S.; Bartsch D.; Schloss P. Antidepressant-induced internalization of the serotonin transporter in serotonergic neurons. FASEB J. 2008, 22 (6), 1702–1714. 10.1096/fj.07-095471. 18216289

[ref58] Talley N. J. SSRIs in IBS: sensing a dash of disappointment. Clin. Gastroenterol. Hepatol. 2003, 1 (3), 155–159. 10.1016/S1542-3565(03)70030-5. 15017485

[ref59] Lynch M. E. Antidepressants as analgesics: a review of randomized controlled trials. J. Psychiatry Neurosci. 2001, 26 (1), 30–36. 11212591 PMC1408040

[ref60] Kułak-Bejda A.; Bejda G.; Waszkiewicz N. Antidepressants for irritable bowel syndrome—A systematic review. Pharmacol. Rep. 2017, 69 (6), 1366–1379. 10.1016/j.pharep.2017.05.014. 29132094

[ref61] Teng S.; Yang Y.; Zhang W.; Li X.; Li W.; Cui Z.; Min L.; Wu J. Antidepressant fluoxetine alleviat*es coli*tis by reshaping intestinal microenvironment. Cell Commun. Signaling 2024, 22 (1), 176 10.1186/s12964-024-01538-5. PMC10935910 38475799

[ref62] Ba D. M.; Yadav S.; Liu G.; Leslie D. L.; Vrana K. E.; Coates M. D. Clinical outcomes associated with antidepressant use in inflammatory bowel disease patients and a matched control cohort. Sci. Rep. 2024, 14 (1), 1060 10.1038/s41598-024-51282-6. 38212393 PMC10784571

[ref63] Eyzaguirre-Velásquez J.; González-Toro M. P.; González-Arancibia C.; Escobar-Luna J.; Beltrán C. J.; Bravo J. A.; Julio-Pieper M. Sertraline and Citalopram Actions on Gut Barrier Function. Dig. Dis. Sci. 2021, 66 (11), 3792–3802. 10.1007/s10620-020-06702-8. 33184794 PMC8510962

